# Circadian transcriptome of pancreatic adenocarcinoma unravels chronotherapeutic targets

**DOI:** 10.1172/jci.insight.177697

**Published:** 2024-05-08

**Authors:** Deepak Sharma, Darbaz Adnan, Mostafa K. Abdel-Reheem, Ron C. Anafi, Daniel D. Leary, Faraz Bishehsari

**Affiliations:** 1 Rush Center for Integrated Microbiome and Chronobiology Research, Rush Medical College, Rush University Medical Center, Chicago, Illinois, USA.; 2 Department of Medicine, Perelman School of Medicine, University of Pennsylvania, Philadelphia, Pennsylvania, USA.; 3 Department of Internal Medicine, Division of Gastroenterology and; 4 Department of Anatomy and Cell Biology, Rush University Medical Center, Chicago, Illinois, USA.

**Keywords:** Oncology, Therapeutics, Bioinformatics, Drug screens, Oncogenes

## Abstract

Pancreatic ductal adenocarcinoma (PDA) is a lethal cancer characterized by a poor outcome and an increasing incidence. A significant majority (>80%) of newly diagnosed cases are deemed unresectable, leaving chemotherapy as the sole viable option, though with only moderate success. This necessitates the identification of improved therapeutic options for PDA. We hypothesized that there are temporal variations in cancer-relevant processes within PDA tumors, offering insights into the optimal timing of drug administration — a concept termed chronotherapy. In this study, we explored the presence of the circadian transcriptome in PDA using patient-derived organoids and validated these findings by comparing PDA data from The Cancer Genome Atlas with noncancerous healthy pancreas data from GTEx. Several PDA-associated pathways (cell cycle, stress response, Rho GTPase signaling) and cancer driver hub genes (*EGFR* and *JUN*) exhibited a cancer-specific rhythmic pattern intricately linked to the circadian clock. Through the integration of multiple functional measurements for rhythmic cancer driver genes, we identified top chronotherapy targets and validated key findings in molecularly divergent pancreatic cancer cell lines. Testing the chemotherapeutic efficacy of clinically relevant drugs further revealed temporal variations that correlated with drug-target cycling. Collectively, our study unravels the PDA circadian transcriptome and highlights a potential approach for optimizing chrono-chemotherapeutic efficacy.

## Abstract 2

Characterization of the pancreatic ductal adenocarcinoma circadian transcriptome suggests potential approaches for optimizing chrono-chemotherapeutic efficacy.

## Introduction

Pancreatic ductal adenocarcinoma (PDA) is the most prevalent neoplastic disease of the pancreas, accounting for more than 90% of all pancreatic malignancies (1). PDA has a 5-year survival rate of only 6%–8% and is the fourth most frequent cause of cancer-related deaths. The incidence of PDA is expected to further rise in the next 10 years, with a 2-fold increase in both new diagnoses and the number of PDA-related deaths (2). Currently, surgical resection and chemotherapy are the common treatment modalities for PDA (3). However, more than 90% of tumors are unresectable at the time of diagnosis and progress further within a few months of chemotherapy (3). These metrics highlight the need for better and more optimized therapeutic options to treat PDA.

Almost all species exhibit circadian rhythms or daily changes in their physiology and behavior (4). These daily rhythms, which evolutionarily developed in response to changes in the cycling environment, could arise from a timekeeping system cued by various signals within the organism. Greater than 50% of the transcriptome is known to exhibit temporal fluctuations in a tissue-specific manner (5). These oscillations are controlled by the central master clock located in the suprachiasmatic nuclei or by noncentral cues (5). At the molecular level, the circadian clock is a transcriptional autoregulatory feedback loop, consisting of activator genes (i.e., *CLOCK*, *NPAS2*, and *BMAL1*) that induce the expression of their own repressors (i.e., *PER1–3*, *CRY1–2*) (6). In a functional clock, the CLOCK-BMAL1 complex binds to the regulatory elements of repressor genes and promotes their transcription and protein production. PER and CRY proteins then translocate to the nucleus, bind to the CLOCK-BMAL1 complex, and repress their own transcription by acting on CLOCK-BMAL1 activity. The CLOCK-BMAL1 complex also induces expression of nuclear receptors (i.e., *NR1Ds*, *RORs*) that regulate *BMAL1* expression, further fine-tuning the clock. This 24-hour cycling of the clock regulates the time-dependent expression of various genes through transcriptional regulatory mechanisms (6, 7).

Like healthy tissues, cancer cells also exhibit rhythmic molecular patterns that may influence tumor progression, proliferation, and development (8), spurring the development of cancer chronotherapy (9). Prior preclinical as well as clinical studies have shown time-dependent variation in drug efficacy of chemotherapeutic drugs, including those used for PDA (10, 11). Despite showing some potential, cancer chronotherapy has not always yielded consistent results. This may be a result of our simplistic experimental approach of randomly assigning individuals to morning or evening drug dosing without considering possible interindividual differences in their tumor biology (12).

Over the past decade, we have learned that tumors of individuals with the same cancer show large molecular differences (13). Oncogenic processes impinge on various cellular pathways, several of which are known to show rhythmic patterns (e.g., cell cycle, DNA replication) and are targetable by chemotherapies (14). Tumor molecular changes may accompany variations in the tumor rhythmic profile, causing interindividual differences in chrono-chemotherapeutic efficacies (10). These issues are further confounded in PDA, with one of the highest interindividual tumor molecular heterogeneities and high rates of ineligibility for surgical resection that limit adequate sample acquisition for molecular profiling.

With the aim to identify and optimize chronotherapeutic targets for PDA, in this work we systematically studied the pancreatic circadian transcriptome. To achieve this, we investigated patient-derived organoids (PDOs) that were generated from the treatment-naive cancer tissue of PDA patients (15). Using PDA data from The Cancer Genome Atlas (TCGA) (16) and noncancerous healthy pancreas data from GTEx (17), we verified that the rhythmicity profiles observed in the PDOs were cancer specific. Transcriptome-wide variations in the temporal transcriptome allowed us to delineate pathways receptive to chronotherapy in a sample-specific manner, which was verified in vitro through time-dependent efficacy testing of top drug candidates. Collectively, this study suggests the existence of a circadian transcriptome in PDA that could help identify potential drug targets to optimize personalized chronotherapy for PDA.

## Results

### Circadian cycling of gene expression and cellular processes in PDOs of PDA.

**Figure 1:**
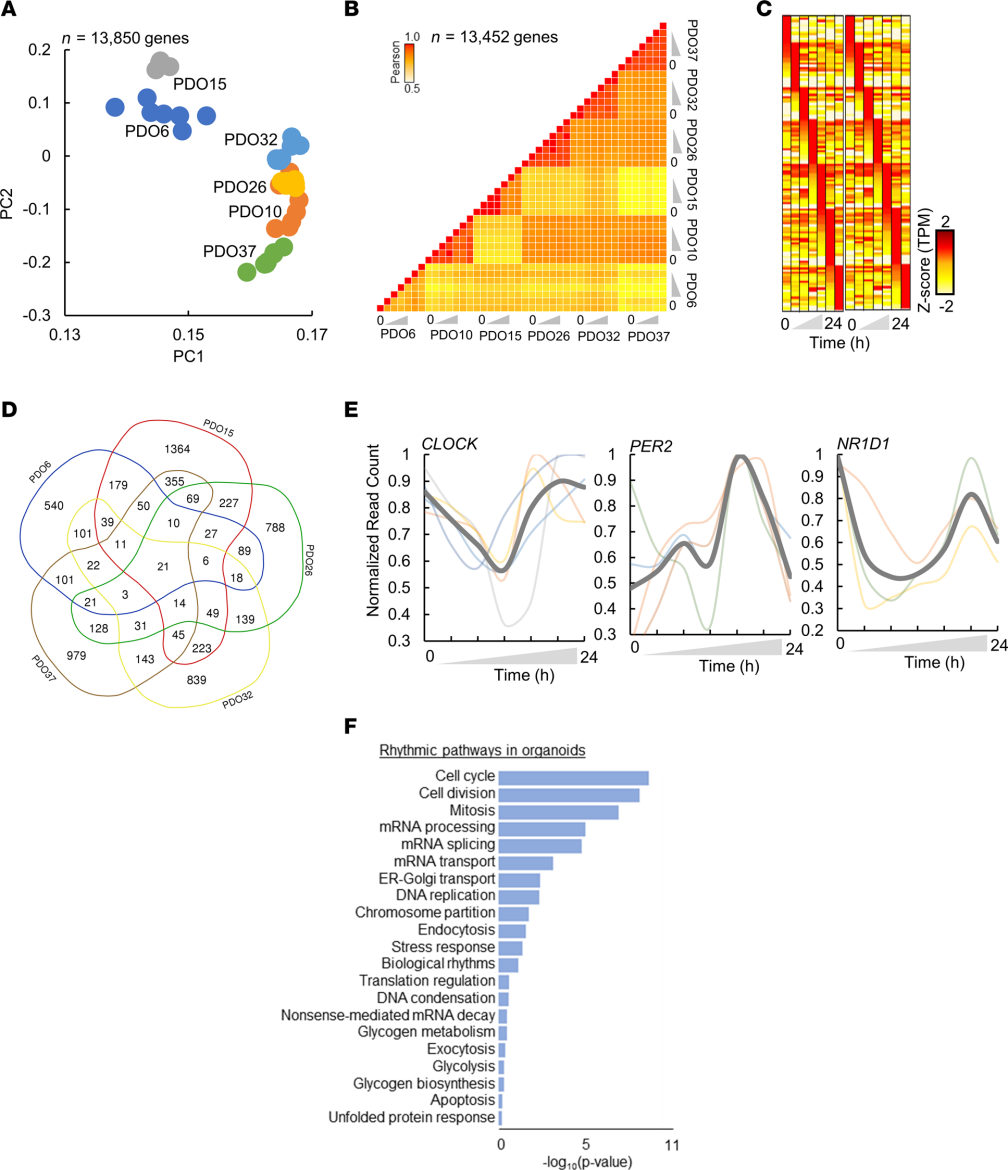
Circadian cycling of gene expression and cellular processes in patient-derived organoids (PDOs). (**A**) Principal component analysis (PCA) of PDOs. Each sample corresponds to 13,850 genes. PDO15, gray; PDO6, darl blue; PDO32, light blue; PDO25, yellow; PDO10, orange; PDO37, green. (**B**) Pearson’s pairwise correlation of normalized expression (TPM) for cell lines at each time point (*n* = 13,850 genes). (**C**) *Z*-scored heatmap of temporal gene expression in PDOs (*n* = 999 genes). *Z* score corresponds to a range of –2 to +2. (**D**) Venn diagram showing rhythmic genes in each PDO. (**E**) Temporal profiles of selected circadian clock genes. Temporal expression of each PDO is shown with transparent lines. Average expression at each time point is shown with a gray solid line. Color code represents PDOs as per **A**. (**F**) The most enriched biological pathways for genes that were rhythmic in PDOs.

With the goal of identifying a circadian transcriptome reflective of PDA, we began by quantifying the temporal gene expression of PDOs from pancreatic cancer patients. Briefly, we generated 6 PDOs through fine-needle biopsies obtained during clinically indicated endoscopic procedures from treatment-naive PDA patients (Supplemental Figure 1; supplemental material available online with this article; https://doi.org/10.1172/jci.insight.177697DS1) (15, 18). Subsequently, we synchronized the PDOs with dexamethasone. After 1 hour, the dexamethasone was replaced with a cell culture medium and cultured for 24 hours before further studies. Following that period, gene expression was quantified over 24 hours at 7 circadian time points (CTs) by performing RNA-seq (depth: >40 million reads per time point). Visualization of the normalized expression of each PDO in the principal component space revealed clustering into distinct organoid subgroups, independent of the collection time (Figure 1A). Despite the observed dissimilarities among PDOs, expression data from different CTs of a given PDO exhibited marked correlations between any 2 CTs of that PDO (*R*^2^ > 0.95, Figure 1B), indicating that gene expression of PDOs show larger differences among patients than the circadian variation within a PDO. Overall, these data confirm variations in gene expression among PDOs and demonstrate temporal transcriptome oscillations within each PDO.

To validate the molecular identity of the PDOs, we compared the average gene expression of PDOs with the expression of well-known cancer driver genes (CDGs, *n* = 736) (19) from PDA samples obtained from TCGA data sets (*n* = 184). The list of CDGs was obtained from the Cancer Gene Census, an ongoing effort to catalog genes with changes causally implicated in cancer formation (19). We observed a marked correlation between PDO and TCGA expression profiles of CDGs (Supplemental Figure 2, A and B), validating the molecular integrity of PDOs as a pancreatic cancer system.

To delineate the circadian transcriptome of each PDO, we defined cycling genes as transcripts exhibiting cosine oscillations (FDR of integrated *P* value ≤ 0.05, Meta2D; ref. 20), with peak expression observed at different CTs (Figure 1C). Rhythmic transcripts were identified in every PDO, with several transcripts common to 2 or more PDOs (Figure 1D). Consistent with the role of the molecular clock in cellular circadian rhythms (21), core circadian clock genes (CCGs) were rhythmic in all the organoids, exhibiting similar amplitude and phases (Figure 1E). Upon selecting for all the genes that were rhythmic in at least one of the organoids, a total of 8.5% (1,142 transcripts) of the PDO transcriptome was found to be rhythmic. To capture oscillations in a larger framework of gene networks biologically relevant to PDA, we analyzed the circadian transcriptome of the PDOs at a pathway level. Pathway enrichment showed significant enrichment for circadian oscillations (*P* ≤ 0.05) in several essential cellular processes (e.g., cell cycle, mRNA processing, DNA replication, stress response) (Figure 1F). Interestingly, the most rhythmic patterns in organoids are related to the cell cycle, a process known to encompass major targets of chemotherapeutic drugs in PDA (22).

### Cancer-associated genes and processes are rhythmic in PDOs.

**Figure 2:**
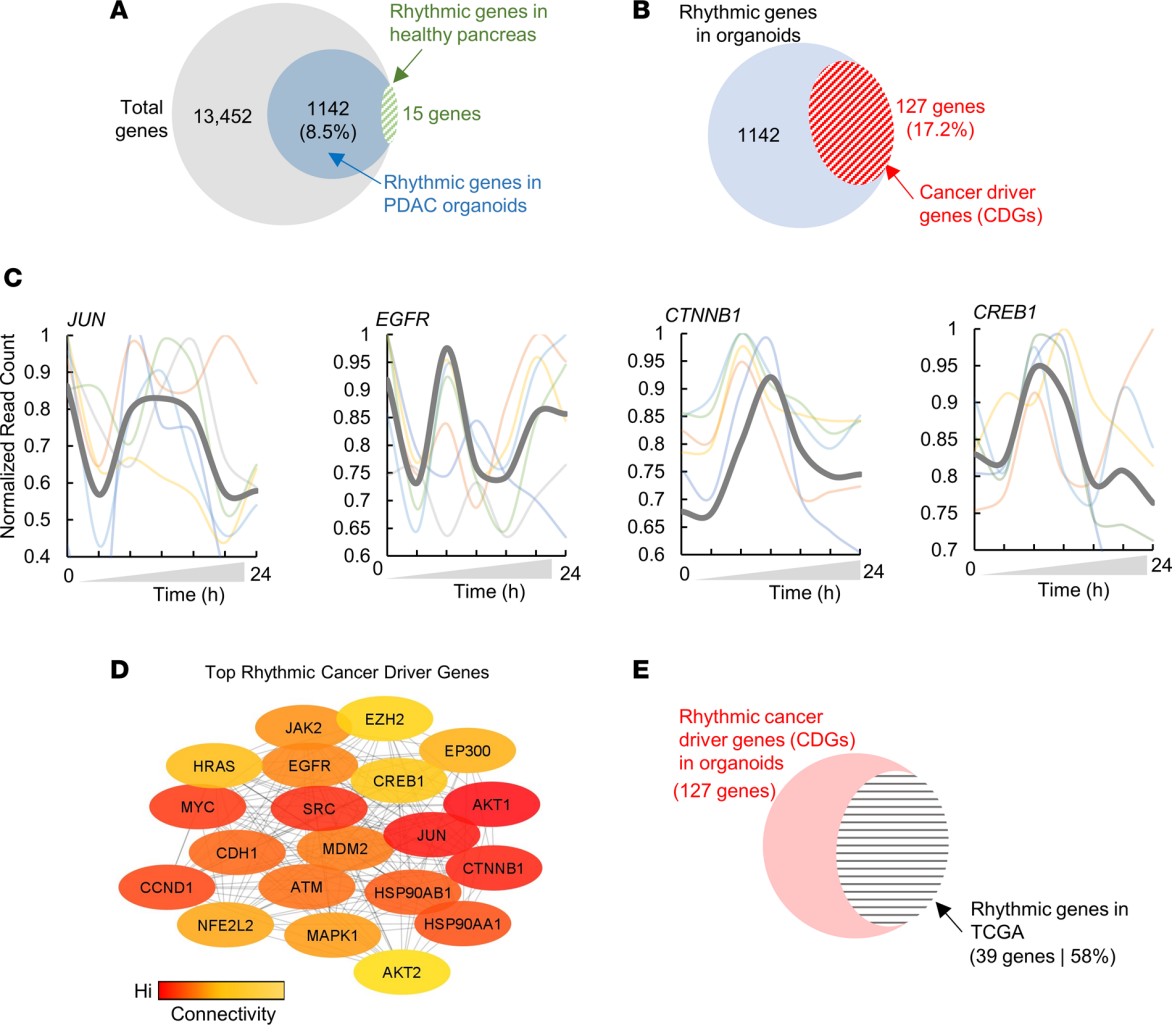
Cancer-associated genes and processes are rhythmic in PDOs. (**A**) Venn diagram of rhythmic genes in PDA organoids (*n* = 1,142) out of total genes in the PDO transcriptome (*n* = 13,452). Genes rhythmic in noncancerous pancreas data sets are also overlapped and shown as green dashed ellipse (*n* = 15). (**B**) Venn diagram of the intersection of rhythmic genes in organoids (*n* = 1,142) and cancer driver genes (CDGs) obtained from the cancer gene census. (**C**) Temporal profiles of selected CDGs that were rhythmic in PDOs. Temporal expression of each PDO is shown with transparent lines. Average expression at each time point is shown with gray solid line. Color code represents PDOs as per Figure 1A. (**D**) Top rhythmic CDGs (r-CDGs) based on the network connectivity parameter (the maximum clique centrality, or MCC). Node color corresponds to MCC value, with red being the highest. (**E**) Venn diagram of the intersection of r-CDGs in PDOs (*n* = 127) and r-CDGs in samples from TCGA (*n* = 183).

To determine whether the rhythmic transcripts of PDOs are cancer specific, we extracted the rhythmic genes of the healthy human pancreas from GTEx data sets, as recently described (23). Interestingly, a comparison of rhythmic genes in the PDOs with those of noncancerous pancreas revealed only 15 genes in common, mainly including the CCGs (Figure 2A and Supplemental Figure 2C), suggesting that the rhythmic genes observed in the pancreatic PDOs are distinct from those in the healthy pancreas.

To examine whether the rhythmic PDO genes are cancer relevant, we intersected the rhythmic transcripts of PDOs (*n* = 1,142) with the CDGs (*n* = 736 as above). A significant proportion of CDGs (*n* = 127 of 736, 17.2%; *P* ≤ 0.05) were among the rhythmic transcripts (termed Rhythmic CDGs or r-CDGs; Figure 2B) in at least one PDO. Among those were several CDGs known to be involved in PDA, including *MYC*, *CCND1*, *SRC*, *AKT1*, *JUN*, *CTNNB1*, *EGFR*, *CREB1*, *MDM2*, and those encoding heat shock proteins (*HSP*s) (24–27), which is consistent with findings from experimental circadian models of carcinogenesis in PDA (28). Temporal profiles of selected CDGs are depicted in Figure 2C.

To assess the functional importance of the r-CDGs, we constructed a protein-protein interaction network using the STRING database (29) and computationally measured node connectivity based on network topology parameters. We chose to compute the maximal clique centrality (MCC) for each node, as this algorithm was found to perform well in inferring the central elements of biological networks (30). We measured the MCC for all the r-CDGs, along with their first-degree neighbors, and ranked the r-CDGs based on the MCC score. Compared with the background, the majority of r-CDGs were identified as high-connectivity nodes (104 of 127 r-CDGs), with 54 r-CDGs computed as hub proteins (i.e., >30 nodes connecting to the r-CDG; Supplemental Figure 3A). Among the r-CDGs with the highest MCC scores were *EGFR*, *MYC*, *CCND1*, *JUN*, *AKT1*, and *CTNNB1* (Figure 2D).

Additionally, Benjamini-Hochberg testing (31) for statistical overrepresentation of r-CDGs in biological pathways showed that several essential processes known to be involved in cancer progression were enriched for r-CDGs (Supplemental Figure 4). Notable among those were NF-κB signaling, cell cycle, mRNA processing, and stress response. Each of these processes was previously shown to be altered in PDA and several other cancers (32–35). Furthermore, several r-CDGs occupied central hub nodes in these processes, further substantiating the functional role of r-CDGs (Supplemental Figure 3, B and C). Taken together, our data show that rhythmicity of CDGs and their enrichment in cancer-associated pathways points toward temporal modulation of these processes in PDOs of pancreatic cancer.

To assess whether these r-CDGs found in the PDOs are reflective of the rhythmic profile of human PDA in general, we time-stamped PDA samples of TCGA (*n* = 183) and computed the rhythmic profile of every gene using the CIRCUST methodology, as recently described (36). The intersection of rhythmic genes from TCGA with r-CDGs exhibited a 58% overlap (38 of 65 rhythmic genes from TCGA), suggesting that findings from the rhythmic profiling of PDO are relevant to human PDA (Figure 2E).

### Temporal expression of CDGs is coupled with core CCG expression.

**Figure 3:**
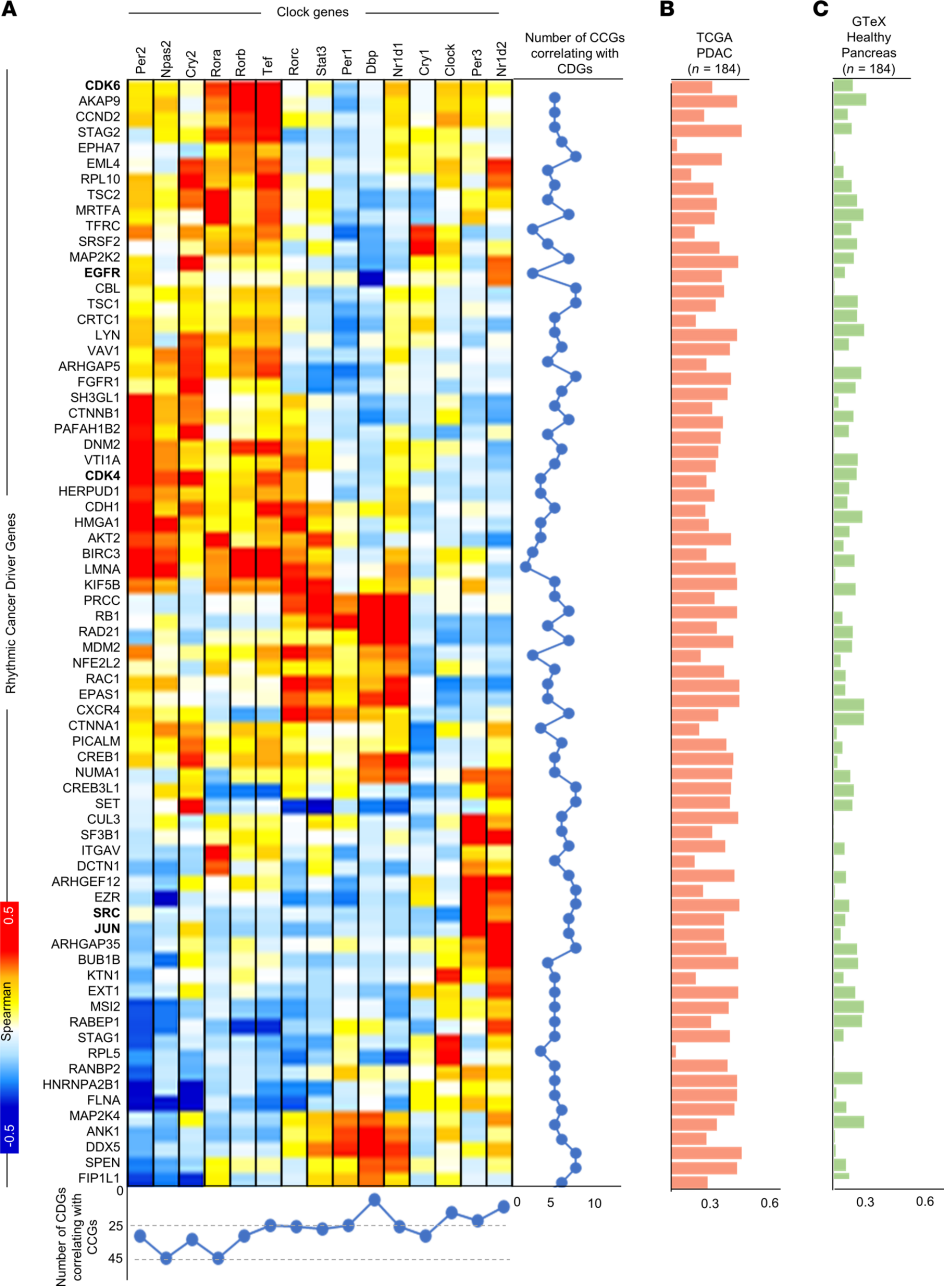
Temporal expression of CDGs is coupled with core circadian clock gene expression. (**A**) Pairwise correlation between CDGs that were rhythmic in PDOs (*n* = 66) and circadian clock genes (CCGs, *n* = 15). CCGs were defined as previously reported (37, 70). Red denotes positive correlation between corresponding CCG and CDG. Blue denotes negative correlation. The total number of CDGs correlating with each CCG are shown in the line plot on the right. The total number of CCGs correlating with each CDG are shown in the line plot at the bottom. (**B**) Each bar represents the trend between the correlation coefficients of pairwise correlation between every CDG and all 15 CCGs in PDOs versus the correlation coefficients of pairwise correlations between each CDG and CCG for PDA samples from TCGA (*n* = 183). (**C**) Each bar represents the trend between the correlation coefficients of pairwise correlation between every CDG and all 15 CCGs in PDOs versus the correlation coefficients of pairwise correlations between each CDG and CCG for noncancerous pancreas samples from GTEx (*n* = 183). Statistically significant correlations in TCGA when compared with GTEx are highlighted in bold. A *P* value (2-tailed *t* test) of less than or equal to 0.05 was considered statistically significant.

CCGs were rhythmic in all the PDOs and represented the only rhythmic pathway in common with those of the noncancerous pancreas (Figure 2A and Supplemental Figure 2C), which is consistent with the conserved role of the circadian clock in maintaining organismal rhythmicity. To test the hypothesis that the rhythmicity of the CDGs (the r-CDGs) of the PDO model is coupled with the CCGs, we examined correlations between the transcriptional expression of r-CDGs (*n* = 127) and CCGs in PDOs (Figure 3A). Based on the literature, the CCGs (*n* = 15) were defined as the genes that control the transcriptional/translational feedback loop of the circadian clock (37, 38). We observed that 117 of 127 (92.1%) r-CDGs have significant correlations (*R*^2^ ≥ 0.3) with at least one clock gene, with several r-CDGs strongly correlating with multiple clock genes (110 of 127, 86.6%). Among the r-CDGs, *EGFR*, *KIF5B*, *TFRC*, and *LMNA* correlated with the maximum number of CCGs (*n* ≥ 8). On the other hand, almost all clock genes (except *PER3* and *CRY1*) correlated with multiple r-CDGs (Figure 3A).

The majority of r-CDGs positively correlated with CCGs (*R*^2^ ≥ 0.6, 68 of 127 r-CDGs) and were enriched for cell cycle, DNA replication, and stress response pathways (Supplemental Figure 5A). For example, cell cycle transcripts *CDK4* and *CDK6* positively correlated with multiple CCGs, with the strongest correlation to *PER2* (*R*^2^ = 0.66) and *RORA* (*R*^2^ = 0.51) respectively. On the other hand, r-CDGs with the strongest negative correlation with CCGs (*R*^2^ > 0.6, *n* = 15) were mainly associated with immune system regulation and mRNA processing pathways (*P* = 5.1 × 10^–3^, Supplemental Figure 5B). Among these, PDA-associated heat shock protein transcripts *HSP90AA1* and *HSP90AB1* negatively correlated with the CCGs *PER2* and *CRY2*, respectively. All in all, we identified 70 strong correlations (at *R*^2^ > 0.6) between CDGs and CCGs in PDOs. These interactions are shown as a network in Supplemental Figure 5C.

To verify whether the CDG-CCG networks observed in PDOs are cancer specific, we performed a similar correlation analysis on PDA samples from TCGA (*n* = 183) and noncancerous pancreas samples from GTEx (*n* = 183). We obtained the profile of every CDG and its correlation with 15 CCGs and compared those correlation coefficients with correlations obtained from the PDOs. We observed a strong linear trend between the correlation profiles of PDO and TCGA data. The trend was markedly weakened when PDOs were compared with GTEx samples (Figure 3B). These data suggest that CDG-CCG temporal correlation profiles of PDOs similarly occur in human PDAs, distinct from noncancerous pancreas.

Since the majority of r-CDGs are clock-coupled, we hypothesized that circadian timing against r-CDGs could affect drug efficacy.

### r-CDGs can inform targets for chronotherapy.

Identification of rhythmicity in genes involved in cancer could pave the way for novel chronotherapeutic strategies (39). Many r-CDGs in our study could be targeted by FDA-approved antineoplastic drugs, as determined through cross-referencing the drug-gene interaction database (40) with the NCI Approved Oncology Set (Supplemental Figure 6). For example, *EGFR* could be a target of several antineoplastic agents. Thus, we postulate that following a circadian timing of drug administration against these r-CDGs may improve efficacy in vitro.

**Figure 4:**
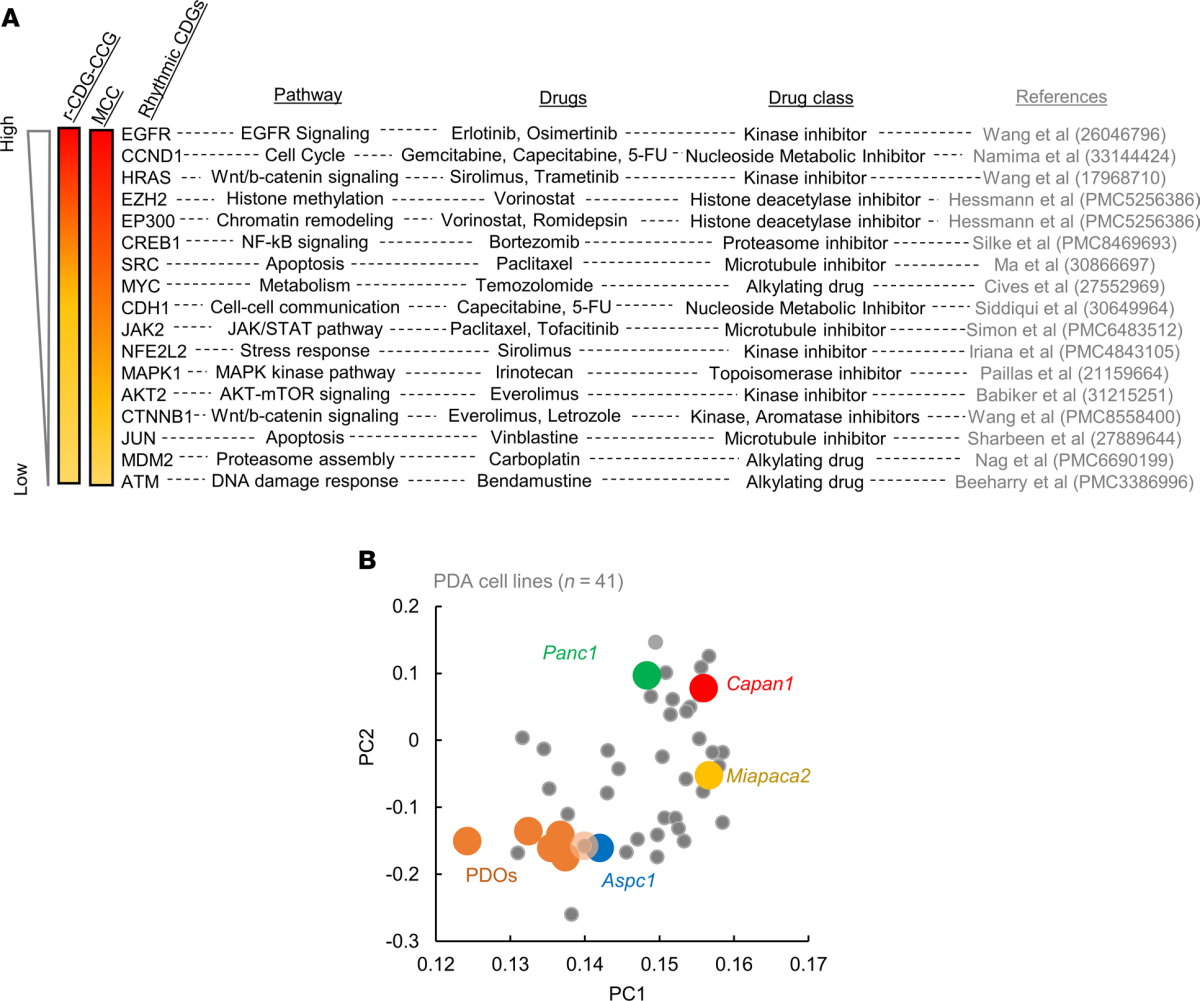
Defining the top chronotherapy targets and in vitro system for testing chronotherapy. (**A**) Top 17 shortlisted r-CDGs ordered based on r-CDG–CCG correlation and network MCC parameter. The pathway and FDA-approved anticancer drugs most associated with each r-CDG are also depicted. Drug classes are provided based on the NCI Approved Oncology Set information as part of the NIH Developmental Therapeutics Program. One reference from an experimental cancer model is listed for each gene-drug combination. (**B**) Principal component analysis (PCA) of 41 PDA cell lines based on the 122 r-CDGs. PDOs are also overlaid on top of the cell line PC space. *Aspc1* was most similar to the PDO in terms of the expression of 122 r-CDGs. *Aspc1*, *Panc1*, *MiaPaca2*, and *Capan1* were selected based on the angle of their rotation vectors in 2 PCs.

First, to identify the best targets for chronotherapy from our list of 127 r-CDGs, we chose genes (*n* = 17 r-CDGs) with the highest network connectivity (Figure 2D). Next, assuming that strong coupling with CCGs could be an indicator of robust rhythmicity (37, 41), we ordered the shortlisted genes based on their r-CDG–CCG correlations. *EGFR* and several cell cycle genes, such as *CCND1*, *CDK4*, and *CDK6*, emerged as top candidates, with erlotinib, gemcitabine, and 5-fluorouracil (5-FU) as potential candidate drugs for chronotherapy (Figure 4A).

To examine the efficacy of our chronotherapy candidates in a well-characterized and controlled system, we turned our attention to human PDA cell lines. In order to select genetically diverse cell lines reflective of both the observed expression pattern of r-CDGs in PDOs and PDA molecular heterogeneity, we matched the steady-state expression levels of 127 r-CDGs in PDOs with 25 well-characterized PDA cell lines from Cell Model Passports (42). By projecting the gene expression onto a 2-dimensional principal component space, we selected 4 molecularly diverse cell lines (AsPC-1, hereafter referred to as *Aspc1*; PANC-1, *Panc1*; MIA PaCa-2, *MiaPaca2*; and Capan-1, *Capan1*), with *Aspc1* being most similar to PDO gene expression (Figure 4B). In addition, the mutation spectrum of PDA-associated genes (42, 43) was distinct among these cell lines (Supplemental Figure 7A), reflective of major molecular subtypes of PDA (44).

**Figure 5:**
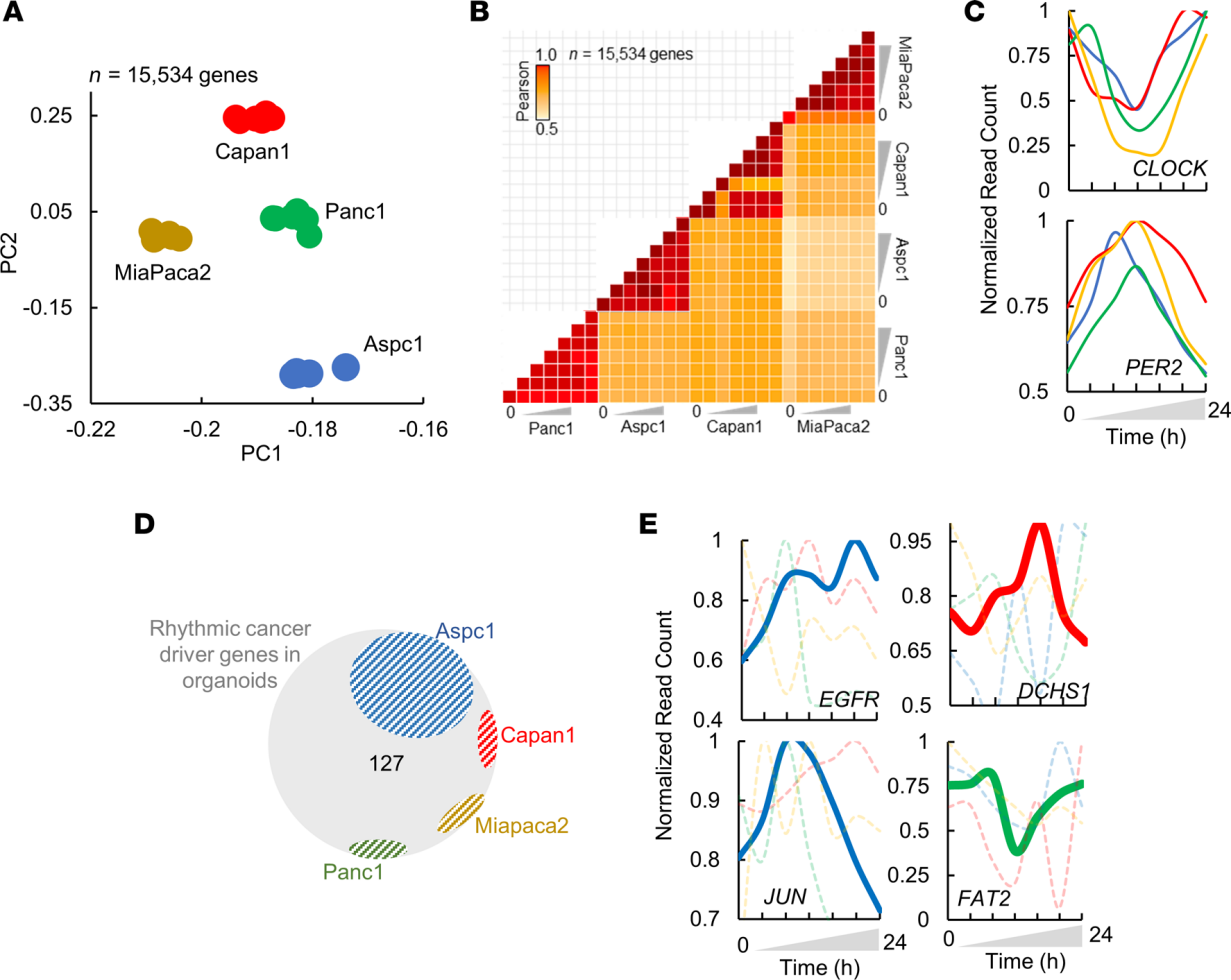
Circadian cycling of gene expression and cellular processes in PDA cell lines. (**A**) Principal component analysis (PCA) of cell lines (*n* = 15,534 genes). *Capan1*, red; *MiaPaca2*, brown; *Panc1*, green, *Aspc1*, blue. (**B**) Pairwise correlation of normalized expression (TPM) for cell lines at each time point (15,534 genes). (**C**) Temporal profiles of selected circadian genes. (**D**) Venn diagram showing intersection of rhythmic cancer driver genes (r-CDGs, *n* = 127) from PDOs and PDA cell lines. (**E**) Temporal profiles of selected cancer-associated genes. Cell lines in which the gene is significantly rhythmic are shown with bold lines. Cell lines in which the gene is not rhythmic are shown with dashed lines in the background. Color code represents cell lines as per **A**.

Like PDOs, we first quantified the temporal gene expression of 4 PDA cell lines over a period of 24 hours using RNA-seq (7 time points; depth: >40 million reads per time point). As expected, we found clear separation of cell lines into distinct groups based on their transcriptome, suggesting the existence of a cell-line-specific gene expression profile (Figure 5, A and B). To delineate the circadian transcriptome of each pancreatic cell line, we defined cycling genes as transcripts exhibiting cosine oscillations (FDR of integrated *P* value ≤ 0.05, Meta2D). Interestingly, most transcripts were nonoverlapping among cell lines (~91%) (Supplemental Figure 7B), possibly due to their molecular diversity. However, like PDOs, CCGs were consistently rhythmic across all cell lines (e.g., *CLOCK* and *PER2*; Figure 5C). The intersection of rhythmic genes in cell lines with the r-CDGs from PDOs showed *Aspc1* with the highest number of genes in common (Figure 5D). Among those were *EGFR*, *JUN*, *CDK4*, and *CDK6*, the same genes found to be rhythmic in PDOs. The time courses for selected rhythmic genes in each cell line are shown in Figure 5E. Furthermore, pathway enrichment analysis of rhythmic genes showed cell-line-specific enrichments in several cancer-associated processes such as cell cycle, membrane trafficking (*Aspc1*), apoptosis (*Panc1*), metabolism of carbohydrates (*MiaPaca2*), and GPCR signaling (*Capan1*) (Supplemental Figure 7C). Many of these pathways were the same as found to be enriched in PDOs (e.g., cell cycle). Taken together, the circadian transcriptome of PDA depicted a cell-line-specific pattern, with several rhythmic cancer-associated processes.

### Time-dependent drug efficacy is associated with the circadian transcriptome of pancreatic cancer cells.

**Figure 6:**
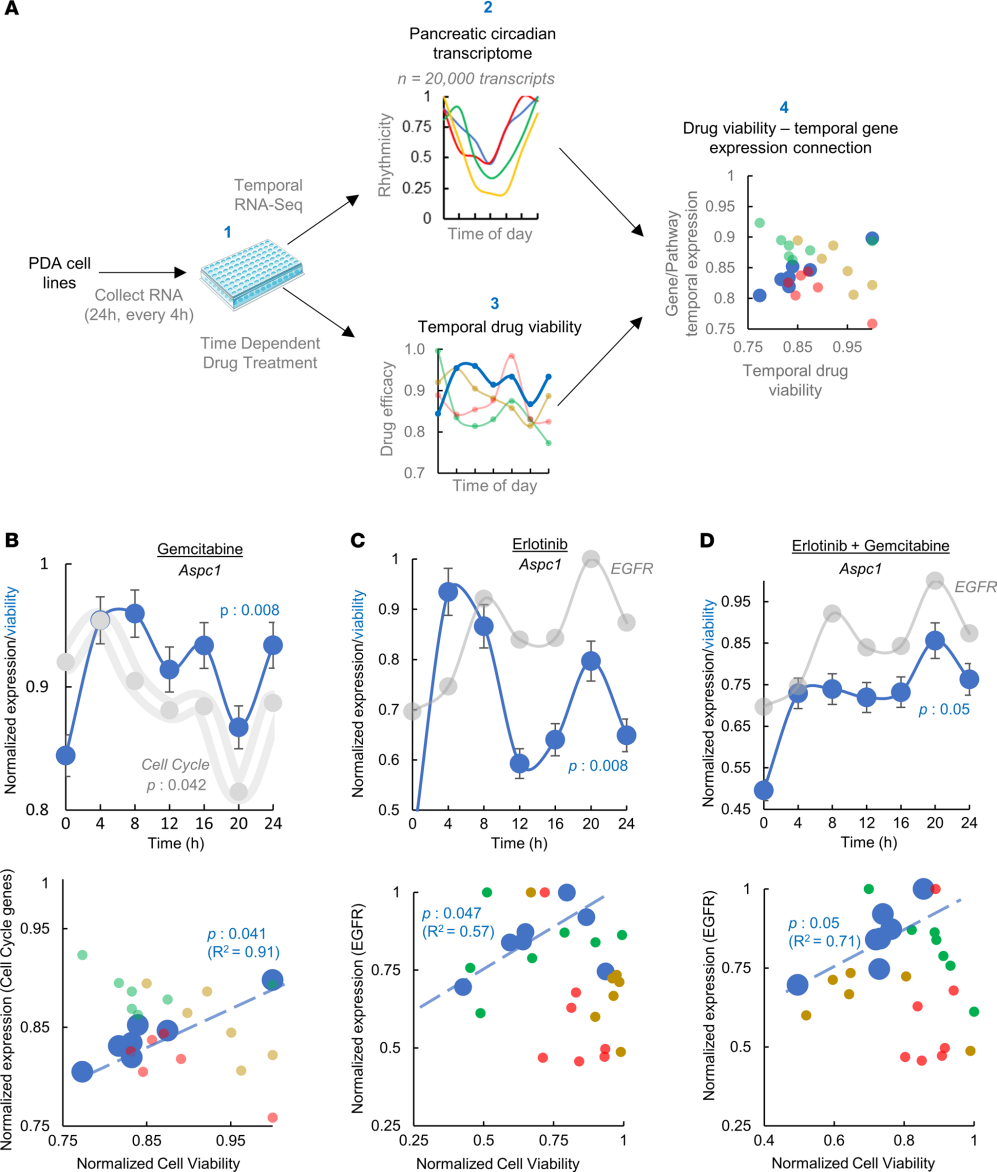
Time-dependent drug efficacy associates with the circadian transcriptome of pancreatic cancer cell lines. (**A**) Framework for elucidation and optimization of chronotherapy for PDA. Pancreatic cancer cell lines were used to perform temporal RNA-seq and time-dependent drug treatment in parallel. Transcriptome-wide circadian transcriptome for all cell lines was quantified, and unique genes and pathways identified reflective of cell line circadian profile. Drug viability was then correlated with the circadian molecular classifiers to identify cell-line-specific chronotherapy drugs. Time-dependent drug efficacy for (**B**) gemcitabine, (**C**) erlotinib, and (**D**) gemcitabine/erlotinib combination is shown. Top panels: Blue lines represent normalized cell viability over time for (**B**) gemcitabine, (**C**) erlotinib, and (**D**) erlotinib/gemcitabine. Gray area in panel **B** represents cumulative expression of cell cycle genes, normalized to the maximum temporal expression for each gene. White line represents median expression levels. Gray lines in panels **C** and **D** reflect normalized expression for EGFR. *P* value indicates 1-sided ANOVA followed by Tukey’s post hoc test to infer significant temporal fluctuations in cell viability (blue) or expression (gray). Bottom panels: Correlation of *Aspc1* cell viability and cell cycle expression (**B**) or EGFR expression (**C** and **D**). *P* value corresponds to the significance of the effect of time of therapy on viability of each cell line. Error bars represent standard deviation from 3 independent experiments.

To investigate whether r-CDGs can be exploited as chronotherapeutic targets, we carried out drug efficacy studies at different time points in the PDA cell lines (Figure 6A). First, we tested the temporal efficacy of the standard PDA chemotherapeutic gemcitabine in PDA cell lines. As a cytidine analog, gemcitabine is incorporated into DNA during replication in the cell cycle and is currently included as an active drug for PDA (45). Furthermore, gemcitabine was previously reported to exhibit diurnal variations in its efficacy (46). Additionally, our analyses identified several cell cycle genes as rhythmic in both PDOs and a specific cell line (i.e., *Aspc1*), thus making gemcitabine an ideal chemo-chronotherapeutic candidate for this study.

Gemcitabine response was estimated by calculating the area under the fitted dose-response curve, where viability indicates fraction of cells alive after treatment (1 = 100% alive). Cell viability measurements over 24 hours (7 drug treatments, 4-hour intervals) showed a strong variation in gemcitabine efficacy in the *Aspc1* cell line (*P* = 8 × 10^–3^), but not in other cell lines (*P* > 0.01, Figure 6B and Supplemental Figure 8A). To assess the relationship between temporal changes in gemcitabine viability in *Aspc1* and its target pathway — cell cycle — we defined a metric for cell cycle rhythmicity in *Aspc1*. For this, we utilized the hub genes that are rhythmic in the cell cycle (see Methods) and obtained a cumulative score (by averaging the expression values at each time point) for the normalized temporal expression for rhythmic hub genes (*n* = 35). The comparison of cell cycle rhythmicity score with gemcitabine efficacy showed a marked similarity in *Aspc1* (*R*^2^ = 0.91, Figure 6B). No significant rhythmic pattern was observed for other cell lines, consistent with an observed cell cycle enrichment of rhythmic genes in *Aspc1* only (Supplemental Figure 7C). Furthermore, the comparison of the composite amplitude of cell cycle transcripts and the amplitude of temporal variation in gemcitabine efficacy showed marked correlation for *Aspc1*, but not for other tested cell lines (*P* = 0.042 for *Aspc1* vs. *P* > 0.05 for *Panc1* as an example, Figure 6B and Supplemental Figure 8B).

In addition to gemcitabine, we tested the temporal efficacy of 5-FU (47). 5-FU is part of the FOLFIRINOX regimen in PDA and acts on the cell cycle to inhibit DNA synthesis by restricting the availability of thymidylate (48). We observed a temporal efficacy pattern for 5-FU in *Aspc1* (*P* = 0.015), but not in other tested cell lines, similar to the pattern we observed for gemcitabine, albeit weaker. However, 5-FU did not show a correlation with the cell cycle expression, probably due to its additional alternative targets and mode of activity (49) (Supplemental Figure 9, A and B). Taken together, these observations highlight the interdependence of cancer cell–specific circadian activity of the transcriptome and temporal drug efficacies of chemotherapeutic agents.

*EGFR* was identified as another top r-CDG in our analysis. Furthermore, *EGFR* is one of the well-known CDGs with a causal role in pancreatic cancer (50). With a goal to investigate the chronotherapeutic potential of a targeted agent based on our transcriptome data, we evaluated temporal cell viability after treatment with erlotinib, an EGFR inhibitor that is used to inhibit cell replication by targeting *EGFR* in pancreatic cancer (51). Interestingly, cell viability upon erlotinib treatment showed a marked temporal variation in *Aspc1* (*P* = 8 × 10^–4^), with a direct correlation to *EGFR* expression by time (*R*^2^ = 0.57, Figure 6C). In addition to *Aspc1*, we also identified a rhythmicity in cell viability for the *Panc1* cell line (*P* = 0.001, Supplemental Figure 10A), which correlated with the *EGFR* temporal expression. Even though *EGFR* was not statistically rhythmic in *Panc1*, it still depicted a significant time-dependent expression pattern (Figure 5E) suggestive of a temporal fluctuation of *EGFR* expression.

Informed by our data on gemcitabine and erlotinib, we proceeded to evaluate the temporal efficacy of gemcitabine plus erlotinib, a combination that has been demonstrated to enhance treatment efficacy in a clinical trial of PDA (51). Similar to the effect of erlotinib alone, we observed a time-dependent efficacy of the combination therapy in *Aspc1* that correlated with *EGFR* temporal expression (*R*^2^ = 0.71, Figure 6D and Supplemental Figure 10B), suggesting that the impact of erlotinib/gemcitabine could depend on time and the levels of *EGFR*.

Altogether, these observations highlight a temporal association between drug efficacy and rhythmic cancer driver genes in pancreatic cancer.

## Discussion

PDA stands out as one of the most lethal cancer types, highlighting the critical need for innovative therapeutic approaches given the scarcity of viable treatment options. To harness the potential of cancer chronotherapy for PDA treatment, in this study, we decoded the genome-wide circadian transcriptome of PDOs and unveiled the circadian landscape of gene expression in PDA. Our work reveals several insights into the potential implication of circadian gene expression in PDA.

First, our analysis uncovered distinct rhythmic patterns in 8.5% of the PDO transcriptome, revealing the existence of a cancer-specific circadian landscape associated with crucial biological processes (Figure 1). Moreover, many of these genes and pathways are known to be critical for PDA progression (Figure 2). Notably, pathways related to cell cycle regulation, mRNA processing, and stress response emerged as focal points. The hallmark of cancer often involves abnormal cell cycle activity, arising from changes in upstream signaling pathways or within genes encoding cell cycle proteins (52). Furthermore, cancer cells employ various stress response pathways, such as the integrated stress response, cytosolic heat shock response, and unfolded protein response (53, 54). Robust rhythmicity in these pathways likely enables cancer cells to navigate environmental stresses, ensure survival, promote proliferation, and maintain cellular fitness during cancer progression.

Second, our study focused on deciphering the circadian transcriptome of PDOs to explore the potential of cancer chronotherapy for PDA. Interestingly, a subset of well-known CDGs exhibited rhythmic expression in PDOs, a finding validated in TCGA samples of PDA. The majority of the r-CDGs were uniquely rhythmic in PDA when compared with noncancerous pancreas samples from GTEx (Figure 2). Additionally, the r-CDGs were strongly correlated with many CCGs, implying a possible mechanistic regulation of CDG temporal expression by the circadian clock (Figure 3). The role of the circadian clock in regulating cancer driver pathways, such as MYC and AKT1, in PDA, has been suggested by experimental models of PDA carcinogenesis (28). This could be supported by the data indicating that various r-CDGs are reported to contain binding sites for clock genes in their promoters (37). The similarities in the rhythmicity profiles of several r-CDGs across patients (Figure 2), coupled with the consistent pattern in their CCG expression (Figure 1), further suggest the involvement of the clock apparatus in modulating CDG transcription. On the other hand, the rhythmic expression profile of certain genes in culture might be independent of the molecular clock and could be associated with components of the media, including the use of glucocorticoids for cell synchronization (55). While this possibility cannot be ruled out, we have identified a significant overlap (~58%) between our PDO-based r-CDGs and the estimated rhythmic genes from human PDA tumors, thus reinforcing the relevance of our findings to human PDA. Altogether, these observations pointed toward a PDA-specific subset of circadian-coupled genes, presenting potential targets for chronotherapy.

The PDOs utilized here can serve as a model system for studying PDA. PDOs propagate in a 3D matrix and recapitulate the histology and transcriptomic/genetic signatures of their tissues of origin, thus providing a unique and reusable tool to identify tumor-specific biomarkers in scarce tissue samples, such as in PDA. Using this model, our study provided several critical insights for PDA chrono-chemotherapy here.

Organoids replicate the molecular architecture (both steady state and circadian) of the original tissue (18, 56), can be repeatedly sampled, and have been shown to depict circadian rhythms (57, 58). The comparison of PDO transcriptomic profiles with TCGA samples revealed marked similarities, emphasizing the relevance of PDOs in recapitulating PDA characteristics. In fact, our analysis revealed that many transcripts, including clock gene transcripts, show temporal changes in their gene expression. The conserved rhythmicity of clock transcripts among different in vitro models (PDOs, PDA cell lines, and human cancer and noncancerous tissues) is consistent with the essential role of the molecular clock in circadian rhythm regulation. Additionally, the majority of r-CDGs and pathways were closely associated with the oscillation of these molecular clock genes, indicating clock-modulated fluctuations, sometimes unique to specific cell lines or PDOs. Hence, in addition to providing a means to study PDA in vitro, PDOs unraveled the individual-specific circadian heterogeneity of PDA.

The PDA-specific circadian dynamics identified in this study could have profound implications for chronotherapy, potentially leading to the identification of personalized chronotherapeutic targets. The findings suggest the development of chronotherapeutic agents tailored to target the PDA-specific circadian transcriptome. As a proof of concept, we showed a direct connection between the temporal gene expression and efficacies of PDA drugs currently in use for chemotherapy (e.g., gemcitabine, erlotinib). Temporal correlation of *EGFR* expression to erlotinib efficacy could be due to the substrate availability and competitive binding of the drug to the *EGFR* tyrosine kinase or other mechanisms, which need to be further studied.

While our observations were primarily made in PDA, they have broader implications and are potentially applicable to other cancers sharing genetic abnormalities with PDA.

### Limitations of the study.

We acknowledge certain limitations in our study. The gene expression data were collected to assess rhythmicity over a 24-hour cycle at 7 time points every 4 hours, which hindered repeated observation of peak-trough rhythmicity and could potentially limit our ability to detect more rhythmic genes. However, we mitigated this by employing a stringent cutoff to define rhythmic genes, using the FDR based on the integrated *P* value of 0.05 or less.

Furthermore, our analysis focused on changes in rhythmicity profiles across cell lines or organoids, considering patterns reflected by the enrichment of biological pathways rather than isolated single genes. This approach safeguards against minor fluctuations in expression counts at specific time points for individual genes that could artificially impact the inference of rhythmicity.

While our in vitro results indicate that matching rhythmic biological pathways with drug targets can optimize drug efficacy, we recognize the need for in vivo verification. Challenges persist in employing appropriate in vivo models due to circadian differences between rodents and humans (59). Nevertheless, we plan to address this by expanding testable features, including additional cells and patient samples. This expansion aims to strengthen our current observations and uncover new patterns, contributing to the construction of a comprehensive model of potential chronotherapeutic targets in PDA. Despite these challenges and the need for further validation, the proof-of-concept work presented in this study can serve as a framework for designing tailored chronotherapeutics based on individual tumor profiles in PDA. 

## Methods

### Sex as a biological variable.

Both male and female patients were included in this study. Sex of the patient did not affect the recruitment, tissue processing, and data analysis.

### Human specimens.

Pancreatic cancer tissue was collected from untreated patients undergoing tissue biopsy at Rush University Medical Center. Immediately following the tissue acquisition, the samples were processed as previously described (18). Briefly, after digestion with collagenase II, Dispase II, and DNase I, biopsy tissues were washed several times and then plated in 50 μL Matrigel domes on a 24-well plate supplemented with 500 μL organoid medium. Organoid growth medium included Advanced Dulbecco’s modified Eagle medium (DMEM)/F12, N2 Supplement, *N*-acetyl cysteine, B27, PGE2, HEPES, nicotinamide, gastrin, hEGF, A83-01, Y-27632, hFGF, and Wnt3A–R-spondin 1–Noggin conditioned media (50% of final volume). Upon culture for a few days, 3D organoids were generated within a Matrigel dome, supplemented with growth factors, and split for growth. Organoids were routinely supplemented with fresh media and mechanically disassociated for expansion.

### Cell lines and culture.

Human pancreatic cell line AsPC-1 (ATCC, CRL-1682TM) was cultured in RPMI-1640 medium (ATCC, 30-2001). PANC-1 (ATCC, CRL-1469TM) and MIA PaCa-2 1 (ATCC, CRL-1420TM) were cultured in DMEM. Capan-1 (ATCC, HTB-79TM) cell line was cultured in Iscove’s modified Dulbecco’s medium (IMDM; ATCC, 30-2005TM). All the cell culture media were supplemented with 10% FBS (except 20% for IMDM), and 1% penicillin-streptomycin, and cells were incubated in a humidified incubator at 37°C with 5% CO_2_.

### IC_50_ value calculation of the chemotherapeutics.

The half-maximal inhibitory concentration (IC_50_ value) was determined by performing a nonlinear regression, dose-response inhibition analysis on normalized data with log-transformed drug concentrations using GraphPad Prism 8. Briefly, compounds were diluted in DMSO via a 5-fold serial dilution from 1,000 to 1.6 μM and further diluted 1:100 in cell line or organoid media to achieve a concentration range of 10 μM to 16 nM. Viability was analyzed using CellTiter-Glo 3D (Promega) cell viability reagent optimized for 3D cultures according to the manufacturer’s instructions, and luminescence was measured on a Synergy HT plate reader (BioTek).

IC_50_ is an indicator of drug efficiency (60). IC_50_ values for gemcitabine, 5-FU, and erlotinib in the cell lines were determined by a cell counting kit (CCK8, Dojindo Molecular Technologies, Inc.) assay and on the PDOs by CellTiter-Glo 3D cell viability assay. The obtained IC_50_ of the drugs was used to evaluate the chronological drug efficiency.

### Time-dependent drug treatment.

The organoids that had reached full development were dissociated into individual cells using TrypLE Express Enzyme (Gibco, 12604021) for a maximum duration of 30 minutes and subsequently filtered through a 40-μm cell strainer. A total of 2,000 cells were seeded in 40 μL of a 20% Matrigel/complete organoid media mixture into the inner wells of a 96-well plate. The cells were then supplemented with an additional 60 μL of complete organoid media. Drug treatment was administered 24 hours following dexamethasone (Sigma-Aldrich) synchronization at 7 Zeitgeber times (ZTs) spanning a 24-hour period. PDOs were treated at the IC_50_ concentrations across different ZTs in triplicate.

### Cell synchronization.

Cell lines and organoids were seeded in 96-well plates and cultured until 70% confluent. For viability in chemotherapy treatment and CCG expression studies, cells were synchronized by incubating with 100 nM dexamethasone for 1 hour. Time point 0 is defined as the time point of treatment to start carried out at 4-hour intervals for 24 hours. After 1 hour, the dexamethasone was replaced with cell culture medium and cultured for 24 hours before further studies.

### Measurement of cell viability.

The viability of cell lines was determined by a cell counting kit (CCK8, Dojindo Molecular Technologies, Inc.) assay according to the manufacturer’s instructions. Briefly, the cells were cultured in a 96-well plate followed by synchronization with dexamethasone treatment. After 24 hours, 10 μL of WST-8 reagent was added to the cells and incubated up to 4 hours, with absorbance measurements at 450 nm using a UV microplate reader (BioTek Synergy HT). Organoid viability was analyzed using CellTiter-Glo 3D optimized for 3D organoids. Reagent (100 μL) was added to the organoids in 100 μL of medium, followed by shaking and incubation for 30 minutes at room temperature, and the luminescence measured on the Synergy HT plate reader.

### RNA extraction and quantitative real-time PCR.

For RNA extraction, cells and organoids were cultured in a 6-well plate followed by dexamethasone treatment. Twenty-four hours after the synchronization, cells were collected and lysed with RLT buffer containing 1% 2-mercaptoethanol (Bio-Rad). According to the instructions, RNA was isolated using an RNeasy extraction kit (Qiagen). RNA concentration was measured using a NanoDrop 2000c spectrophotometer (Thermo Fisher Scientific). cDNA was prepared from the purified RNA using a cDNA Reverse Transcription Kit (Applied Biosystems).

### RNA-seq.

To capture the transcriptomic signatures, RNA was extracted from cell lines and organoids at the time of multiplex analysis. RNA quality and quantity were measured using an Agilent 4200 Tapestation with the High Sensitivity RNA ScreenTape System (Agilent Technologies). Library preparation was completed using the SMARTer Stranded Total RNA-Seq Kit v2 (Takeda). Libraries were sequenced on an Illumina NextSeq 500 instrument using a NextSeq 500 High Output reagent kit (Illumina Inc.) (1 × 75 cycles), with a target read depth of approximately 30–40 million aligned reads per sample. RNA-seq read quality was quantified using FastQC (https://www.bioinformatics.babraham.ac.uk/projects/fastqc/).

### RNA-seq data preprocessing.

Reads from the RNA-seq data were aligned to the *Homo* *sapiens* genome assembly GRCh38 (mm10) using STAR software (61). Duplicated aligned reads were marked and removed using Picardtools software (62). The gene expression count data were extracted using HTseq software (63). The raw count data were normalized, followed by log_2_ transformation. We filtered out genes with mean read counts of less than 6. All data preprocessing was performed using R software (https://www.r-project.org/).

### Principal component analysis.

A data matrix (X) with the normalized transcript expression (transcripts per million, TPM) values for the cell lines or organoids was generated. Data were centered and scaled and a covariance matrix for the transcript expression was calculated. This covariance matrix was used to calculate eigenvectors and eigenvalues, as described previously (64). Eigenvalues were sorted in descending order and K largest eigenvalues were selected (in our case, K = 2 for Figures 1A and 2B). A projection matrix (W) was created from the selected (K) eigenvalues through orthogonal transformation of the original data set (X) to obtain a K-dimensional feature subspace Y. The proportion of variance, cumulative variance, factor loadings, and eigenvalues explained by each component were recorded and the first 2 principal components were plotted. Principal component analysis was conducted in R v.4.3.0 using the *prcomp* 3.6.2 function.

### Multiple correlation analysis.

For each time point for the cell lines and organoids, pairwise comparison of normalized read counts (TPMs, *n* = 15,534 genes) was performed using the *reshape2* library in R. Pearson’s correlation coefficients were plotted using the *ggheatmap* library in R v.4.3.0. Transcripts with TPMs of less than 0.01 or no expression value in more than 2 replicates were removed before analysis.

### Molecular signatures for cell lines.

Molecular signatures for the CDGs corresponding to the 4 PDA cell lines were obtained from Cell Model Passports (42). The compilation of CDGs is an ongoing effort to catalog those genes that contain mutations that have been causally implicated in cancer and explain how dysfunction of these genes drives cancer. The content, the structure, and the curation process of these genes was previously described (65).

### Circadian expression analysis.

The Meta2D cycling algorithm was used to identify genes with a rhythmic signal in our gene expression time-series data (20). MetaCycle:meta2d in R v.4.3.0 was run with the default settings and period lengths (minper = 20, maxper = 28), as described in version 1.2.0 (https://CRAN.R-project.org/package=MetaCycle). The data frame containing normalized expression values (TPM) for each sample was employed as an input. Genes were considered cycling if the combined *P* value was 0.05 or less (the meta2d_pvalue parameter, corresponding to the *P* values of all 3 algorithms incorporated in Meta2D — ARSER, JTK_CYCLE, and Lomb-Scargle).

### Identification of differentially rhythmic transcripts.

Transcripts differentially rhythmic among cell lines or organoids were identified by comparing the FDR of the integrated *P* values (the meta2d_pvalue parameter) obtained from Meta2D across all the conditions. Period was set as default. Total number of rhythmic transcripts in a condition were quantified by counting all the transcripts with an FDR (of the integrated *P* value) of 0.05 or less (Figure 1D and Figure 4C). A transcript with an FDR of 0.05 or less in multiple conditions was defined as rhythmic in multiple conditions. A transcript was defined as differentially rhythmic in a given condition if its FDR of the integrated *P* value was significant in one condition and not in another.

### Pathway enrichment analysis.

All the transcripts with an FDR (integrated *P* value) of 0.05 or less in each condition were utilized for pathway enrichment analysis. Pathway enrichment analysis for rhythmic transcripts was performed with Reactome (66) using a hypergeometric statistical test and Benjamini-Hochberg FDR correction (67). Before the analysis, redundant pathway terms were merged to create a parent term. Pathways containing less than 10 or more than 250 transcripts were excluded. Pathways significantly (*P* ≤ 0.05) enriched for rhythmic genes in each condition were clustered using unsupervised clustering in R v.4.3.0 (Figure 1F and Figure 2F).

### ANOVA.

One-way ANOVA was calculated in R using car v.3.0.10. Mean square differences between and within groups were calculated. Obtained *F* values were compared with the critical value in the *F* table to obtain *P* values. Intergroup differences were significant (*P* < 0.05) when the *F* value exceeded the critical *F* value for the given degrees of freedom.

### Identification of rhythmic hub genes.

Hub genes were identified using 12 network parameters, as implemented in Cytoscape (68): eccentricity, clustering coefficients, DMNC, bottleneck, radiality, MNC, degree, EPC, closeness, betweenness, stress, and MCC. All 12 network parameters for all the genes belonging to a pathway were obtained. Pathways were defined using Reactome knowledgebase annotations (66). Hub genes were defined as those with top 5% values for 9 of 12 network parameters. Rhythmic hub genes for cell cycle were selected when a hub gene had an FDR (integrated *P* value) of 0.05 or less. All the shortlisted hub genes exhibited a similar temporal expression pattern in *Aspc1*.

### TCGA data.

TCGA data were accessed using cBioPortal (69). mRNA expression data and clinical data of 65 PDA samples were obtained from TCGA pancreatic cancer cohort (TCGA-PAAD) database (https://portal.gdc.cancer.gov/ Accessed April 4, 2023.).

To quantify the extent to which TCGA data sets with or without rhythmicity-associated mutations (RAMs) diverge from the cell cycle reference, cell cycle coexpression matrices were generated, and the pairwise distance was computed for TCGA data sets with or without RAMs relative to the reference cell cycle matrix (Δ pairwise distance parameter).

### Statistics.

Statistical analysis was performed using the R program for statistical computing. Data are presented as the mean ± SD. Intergroup comparisons were evaluated with the 2-tailed *t* test, while multiple groups were compared using a 1-way ANOVA, followed by Tukey’s post hoc test. A *P* value of less than or equal to 0.05 was considered statistically significant.

### Study approval.

All pancreatic cancer tissue was collected from patients undergoing tissue biopsy at Rush University Medical Center as previously described (18). The consent of the patients was obtained prior to the biopsy using a study protocol (no. 16071904) approved by the Institutional Review Board at Rush University Medical Center, Chicago, Illinois, USA.

### Data availability.

All the next-generation sequencing data re available in the NCBI Gene Expression Omnibus database (GEO GSE262627). Raw data that support the conclusions of the manuscript and supplemental material are provided in the Supporting Data Values XLS file. R codes for generation of plots, statistical analysis, as well for calculation of rhythmicity are available at https://github.com/deebratforlife/Circadian-pancreatic-cancer/

## Author contributions

DS analyzed data, generated figures, interpreted data, and drafted the manuscript. DA performed experiments, developed methodology, and interpreted data. MAR and DL performed experiments. FB conceptualized the study, acquired funding, developed methodology, provided project administration and supervision, interpreted data, and wrote and revised the manuscript. All authors approved the final manuscript.

## Supplementary Material

Supplemental data

Supporting data values

## Version 1 05/08/2024

Electronic publication
