# Supplementary material for: Circadian transcriptome of pancreatic adenocarcinoma unravels chronotherapeutic targets
Source: JCI Insight. 2024 May 8;9(9):e177697. doi: 10.1172/jci.insight.177697 (PMC11141942; doi:10.1172/jci.insight.177697)
Supplement: Supplemental data [file jciinsight-9-177697-s072.pdf]

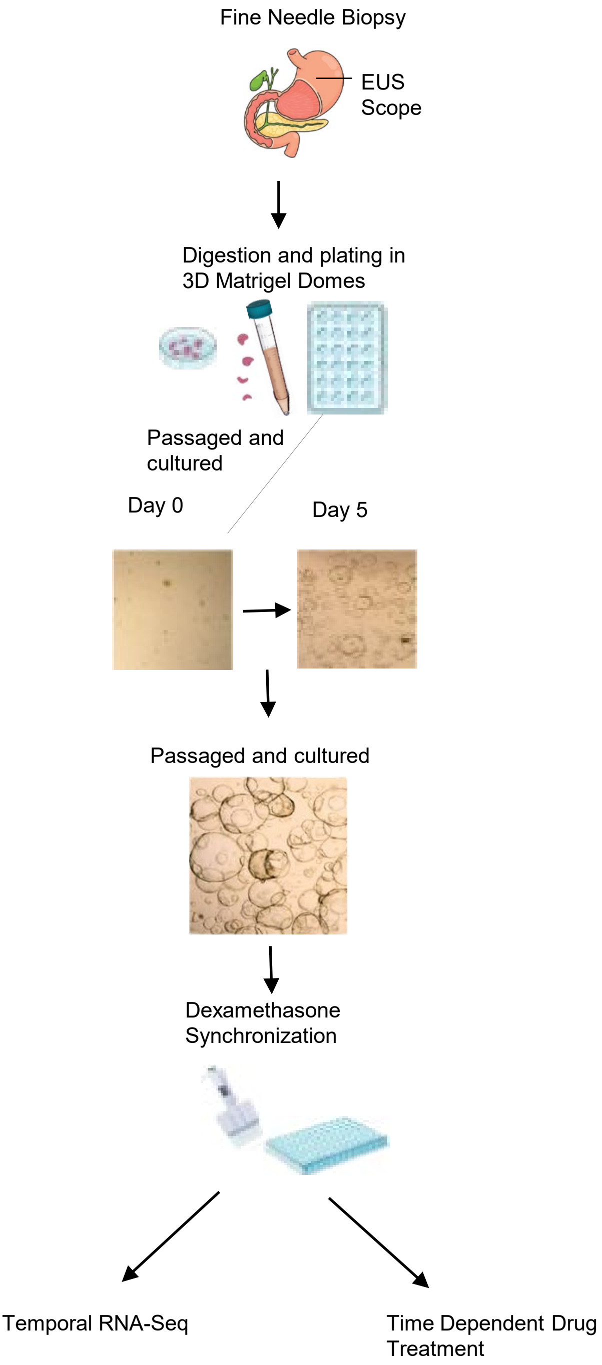

Supplementary Figure 1: Workflow describing the generation, characterization, and multiplex-drug screening assay of PDOs from EUS FNA biopsies. Temporal RNA-Seq and timed drug treatments were carried out in parallel to identify chronotherapeutic targets.

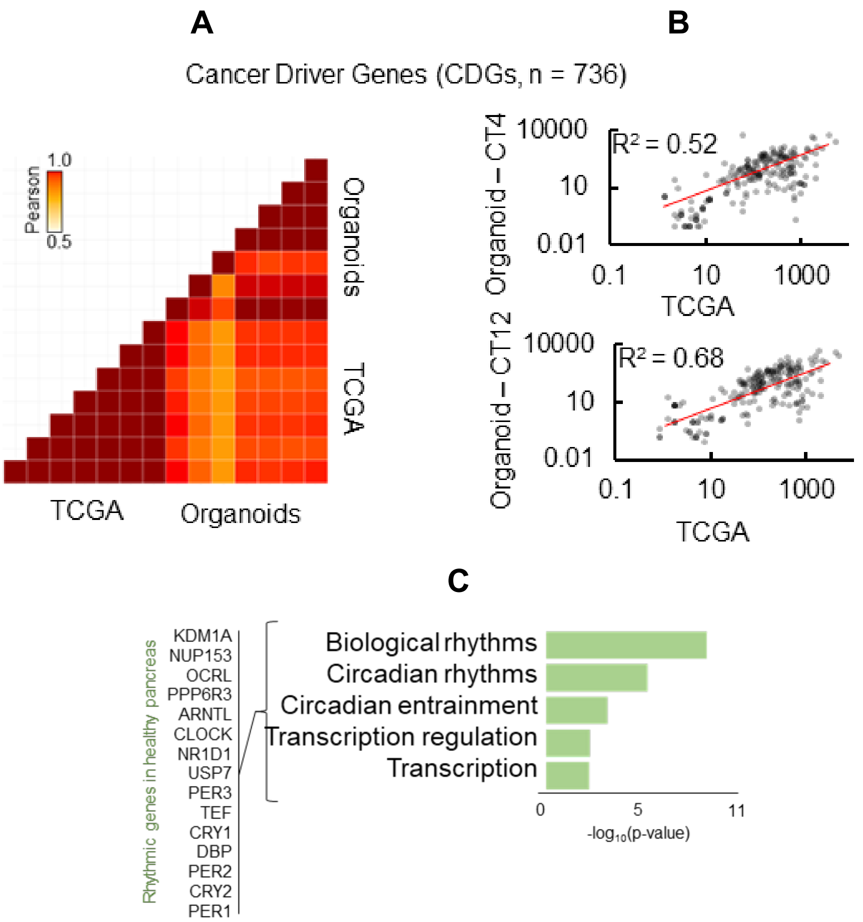

Supplementary Figure 2: Validation of pancreatic derived organoid (PDOs) gene expression pattern. A) Comparison of normalized read counts (TPMs) from PDOs and selected TCGA datasets (n = 7). Note the high pairwise correlation among all the samples. B) Scatter plot of normalized read counts (TPMs) from organoids at specific time points and TCGA datasets (average TPM from n = 7). C) (Left) List of 15 genes that were rhythmic in both PDOs and healthy pancreas. (Right) Pathway enrichment of genes that were rhythmic in both PDOs and healthy pancreas.

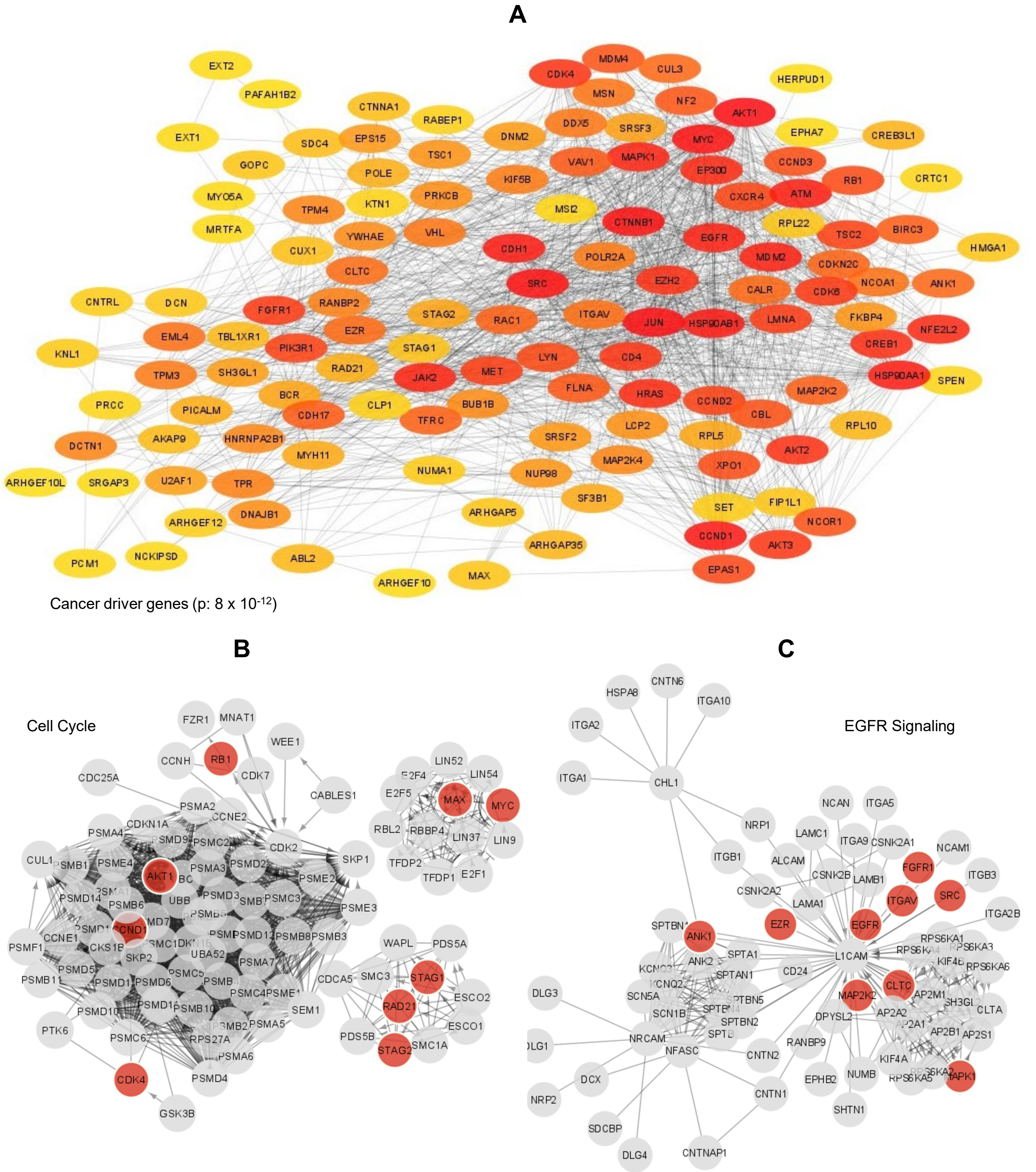
A
EXT2
PAFAH1B2
EXT1
SDC4
GOPC
MYO5A
MRTFA
CTNNA1
RABEP1
EPS15
TSC1
POLE
KTN1
PRKCB
TPM4
YWHAE
VHL
CUX1
CLTC
DNM2
DDX5
SRSF3
VAV1
MAPK1
KIF5B
MSI2
CDK4
MDM4
CUL3
MSN
NF2
AKT1
MYC
EP300
HERPUD1
EPHA7
CREB3L1
CCND3
RB1
CRTC1
CXCR4
ATM
CTNNB1
RPL22
EGFR
CDH1
POLR2A
TSC2
BIRC3
HMGA1
MDM2
CDKN2C
SRC
EZH2
CALR
CDK6
NCOA1
ANK1
CNTRL
DCN
FGFR1
RANBP2
STAG2
RAC1
ITGAV
JUN
HSP90AB1
LMNA
FKBP4
NFE2L2
EML4
TBL1XR1
EZR
PIK3R1
STAG1
CREB1
KNL1
RAD21
JAK2
MET
LYN
CD4
HSP90AA1
SPEN
TPM3
SH3GL1
BCR
FLNA
HRAS
CCND2
MAP2K2
PRCC
PICALM
CLP1
BUB1B
CDH17
TFRC
CBL
RPL10
DCTN1
AKAP9
HNRNPA2B1
SRSF2
LCP2
RPL5
AKT2
MYH11
MAP2K4
XPO1
ARHGEF10L
SRGAP3
U2AF1
TPR
NUMA1
NUP98
SF3B1
SET
FIP1L1
ARHGEF12
DNAJB1
ARHGAP5
NCOR1
CCND1
AKT3
ARHGAP35
PCM1
NCKIPSD
ABL2
MAX
EPAS1
ARHGEF10
Cancer driver genes (p: 8 x 10-12)
B
Cell Cycle
FZR1
MNAT1
WEE1
CCNH
RB1
CDK7
CDC25A
CABLES1
PSMA2
CCNE2
CDKN1A
PSMA4
SMD9
PSMC2
CDK2
CUL1
PSMB1
PSME4
AKT1
PSMA3
PSMD2
SKP1
PSME2
PSMD14
UBB
PSMD3
PSMC3
PSMB6
PSME3
CCND1
CCNE1
PSMF1
CKS1B
SKP2
PSMD12
PSMB8
PSMB3
UBA52
PSMA7
PSMD5
PSMB11
PSMD1
PSMD6
PSMC4
PSME1
SEM1
PSMD10
PSMB10
PTK6
PSMB2
PSMA5
PSMC6
RPS27A
PSMA6
CDK4
PSMD4
GSK3B
LIN52
E2F4
LIN54
E2F5
MAX
MYC
RBL2
RBBP4
LIN37
LIN9
TFDP2
TFDP1
E2F1
WAPL
PDS5A
CDCA5
SMC3
STAG1
ESCO2
RAD21
PDS5B
ESCO1
STAG2
SMC1A
C
EGFR Signaling
HSPA8
CNTN6
ITGA10
ITGA2
ITGA1
CHL1
NRP1
NCAN
ITGA5
LAMC1
ITGA9
CSNK2A1
NCAM1
ITGB1
ALCAM
CSNK2B
LAMB1
FGFR1
ITGB3
CSNK2A2
ITGAV
SRC
LAMA1
ITGA2B
EZR
EGFR
ANK1
RPS6KA1
L1CAM
RPS6KA3
ANK2
SPTA1
KIF5B
RPS6KA6
CD24
SPTAN1
DLG3
SCN5A
MAP2K2
CLTC
AP2M1
SPTBN5
SCN1B
SH3GL2
CLTA
SPTBN2
DPYSL2
AP2A2
AP2A1
AP2B1
AP2S1
NRCAM
RANBP9
NFASC
CNTN2
KIF4A
DCX
EPHB2
NUMB
RPS6KA5
RPS6KA2
NRP2
CNTN1
SHTN1
SDCBP
DLG4
CNTNAP1

Supplementary Figure 3: A) Interaction network of rhythmic cancer driver genes based on the network connectivity parameter (the maximum clique centrality or MCC). Node color corresponds to MCC value, with red being the highest. B) Network of cell cycle with rhythmic cancer driver genes red color coded. C) Network of EGFR signaling with rhythmic cancer driver genes red color coded.

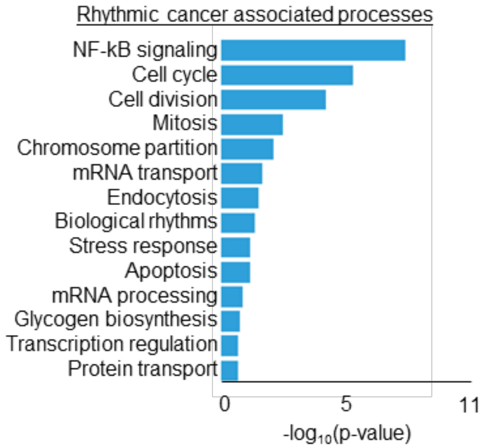

Supplementary Figure 4: Most enriched biological pathways for rhythmic cancer driver genes (r-CDGs, n = 127) in PDOs.

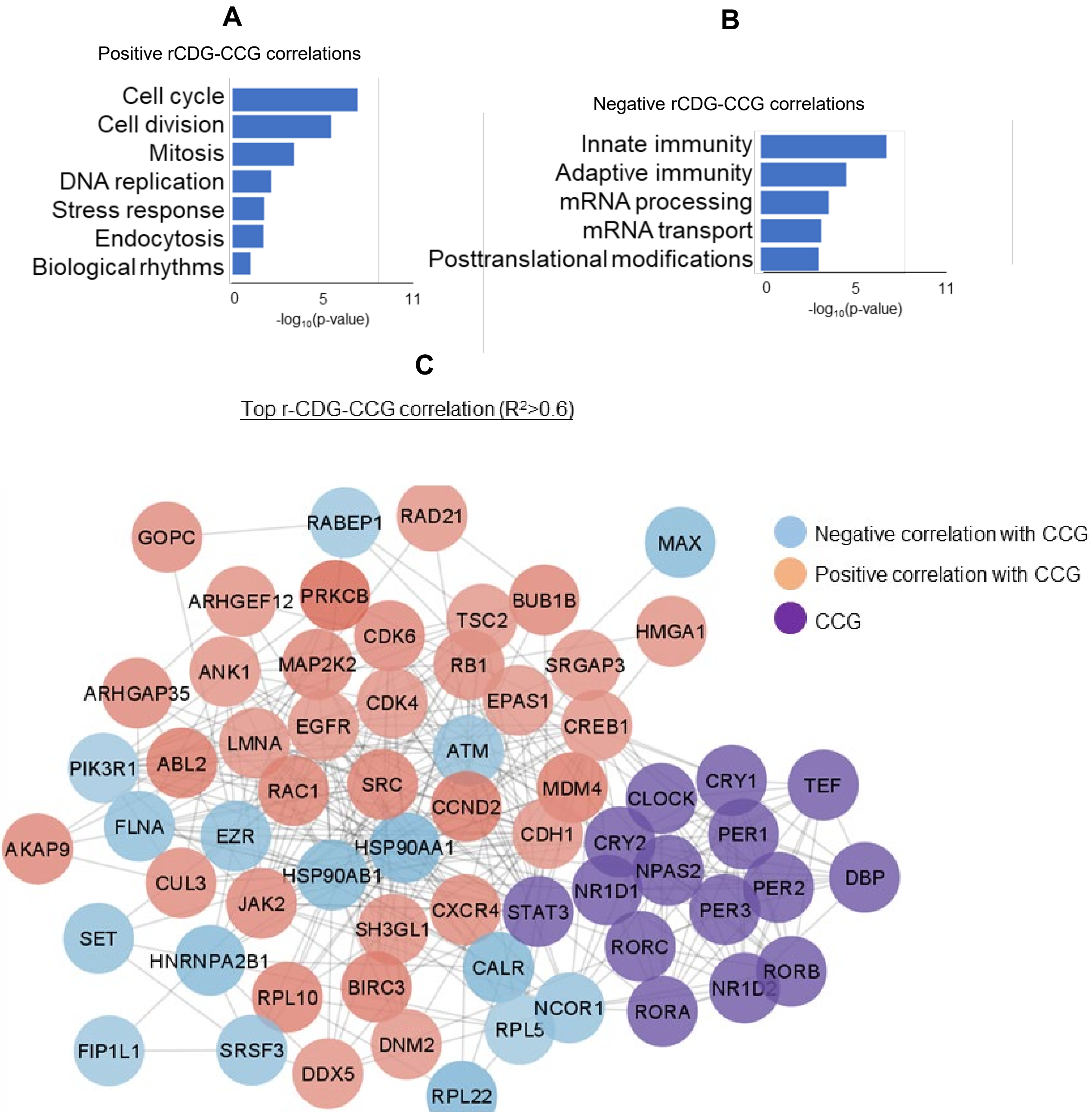

Supplementary Figure 5: Most enriched biological pathways for rhythmic cancer driver genes that (A) positively and (B) negatively correlated with the circadian clock associated genes. C) Network of top r-CDGs and CCGs with $R^2 > 0.6$.

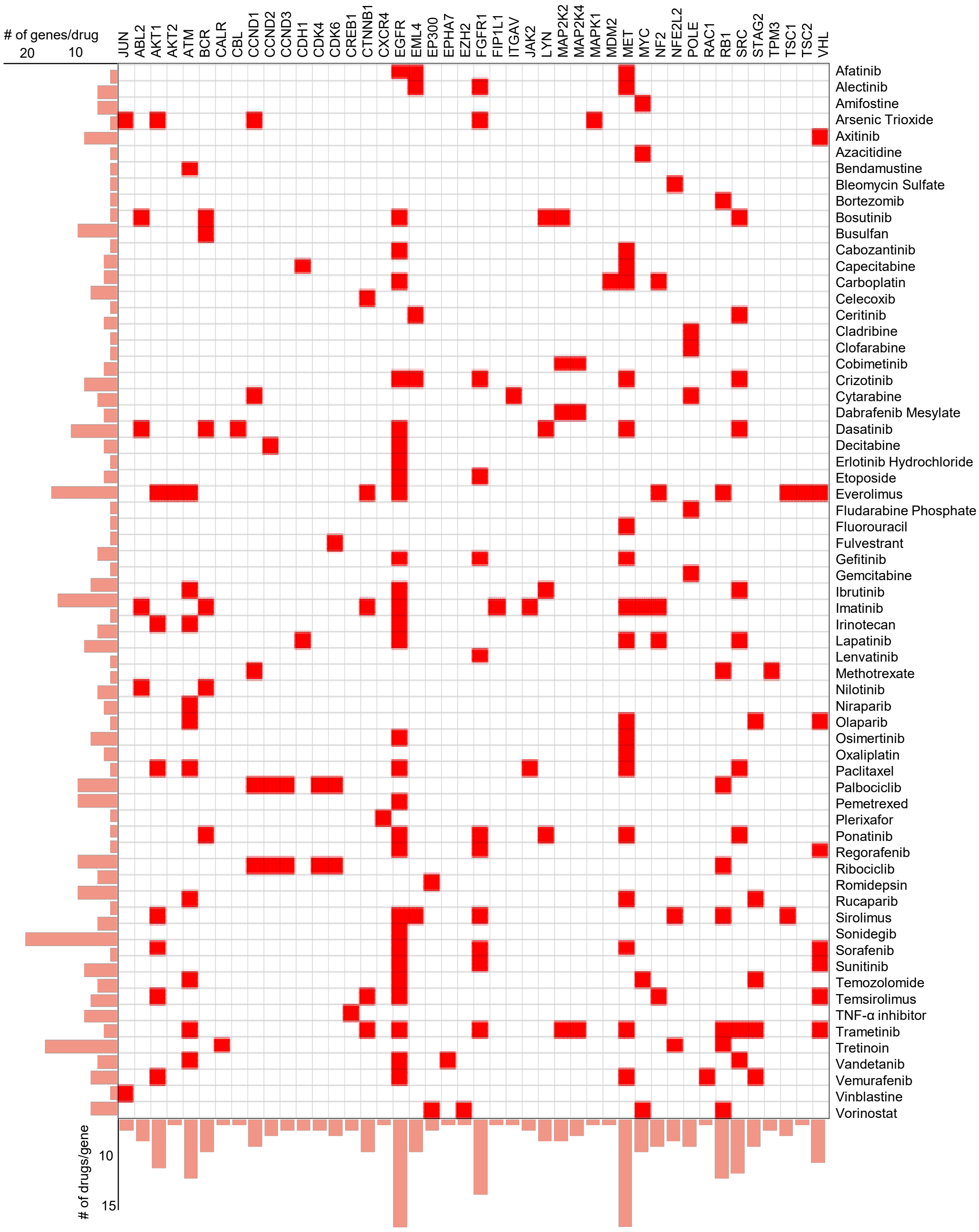

Supplementary Figure 6: Known FDA-approved anticancer drugs against top rhythmic cancer driver genes (r-CDGs) that could be potential chronotherapy targets. Bars represent number of drugs known for each r-CDG. Top drugs in each category are labeled on top of the bars.

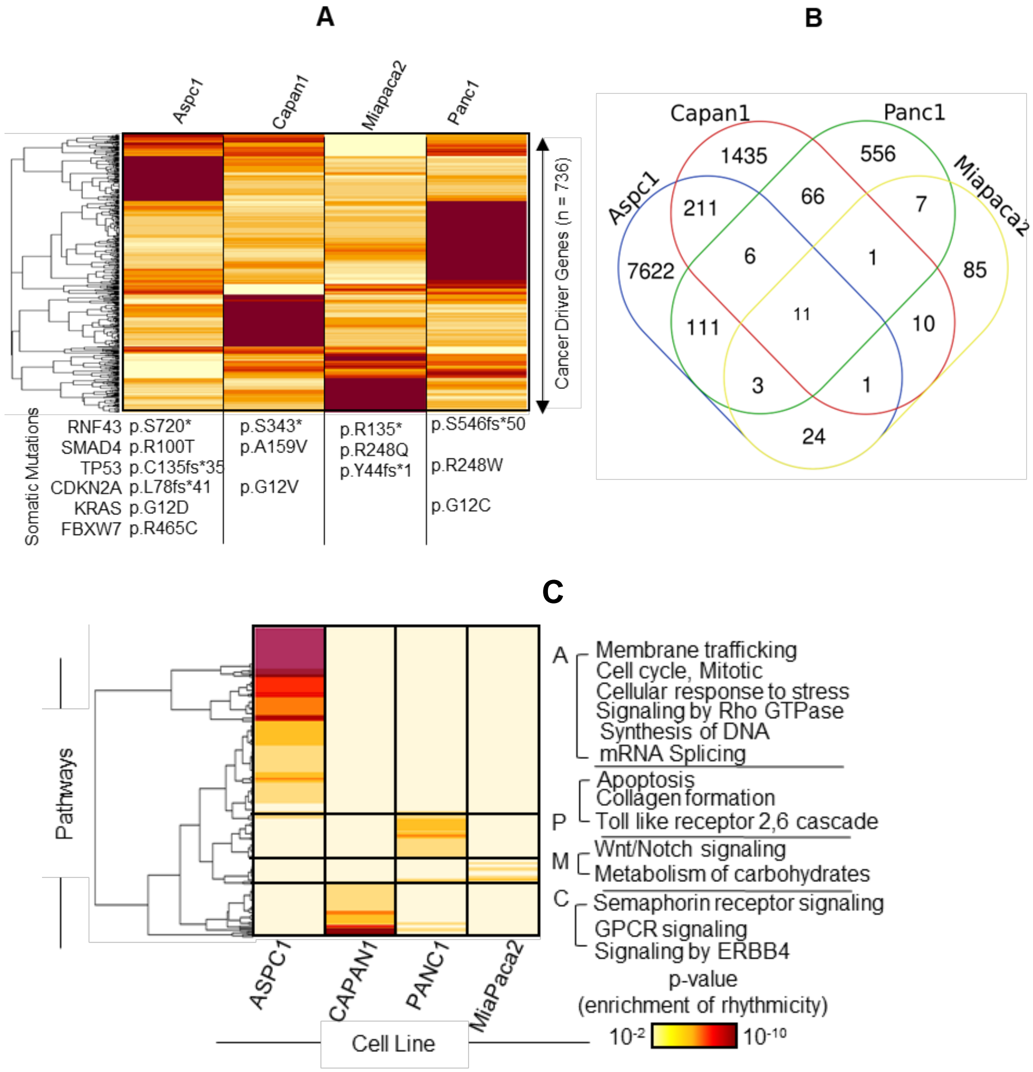

Supplementary Figure 7: B) Molecular signatures for the PDA cell lines used in this study, reflecting the two most common PDA molecular subtypes: squamous (PANC1 and MIAPACA2) and progenitor (PANC1 and MIAPACA2) subtypes (71, 72). Heatmap describes the normalized expression levels (TPM) obtained from cell model passports database. Genes below the heatmap reflect cancer driver genes (CDGs) with cell line specific somatic mutations. ASPC1 and CAPAN1 closely resemble the progenitor subtype, while mimics the squamous subtypes B) Venn diagram showing rhythmic genes in each cell line. C) Enrichment of rhythmic genes in biological pathways in each cell line.

A

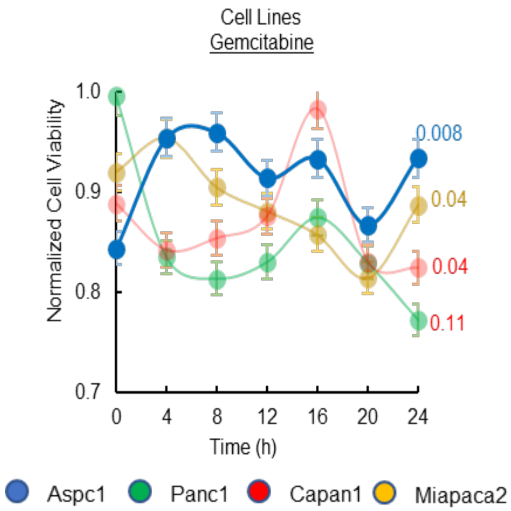

B

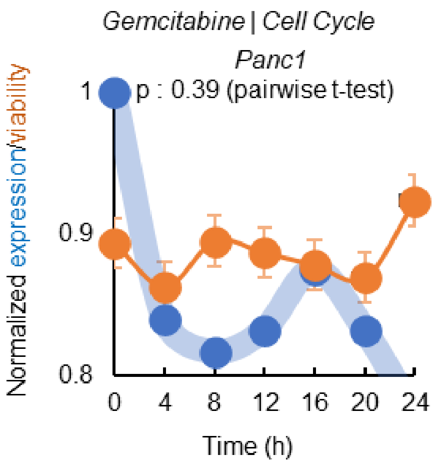

Supplementary Figure 8: A) Temporal drug efficacies for Gemcitabine in 4 cell lines. Colors represent the cell lines. Y-axis is the normalized cell viability for each line. Each datapoint represents an average of 3 replicates. B) Comparison of the amplitude of composite gene expression of cell cycle hub genes and gemcitabine efficacy in PANC1 cell line. p-value corresponds to the significance of the effect of time of therapy on viability of each cell line. Error bars represent standard deviation from 3 independent experiments.

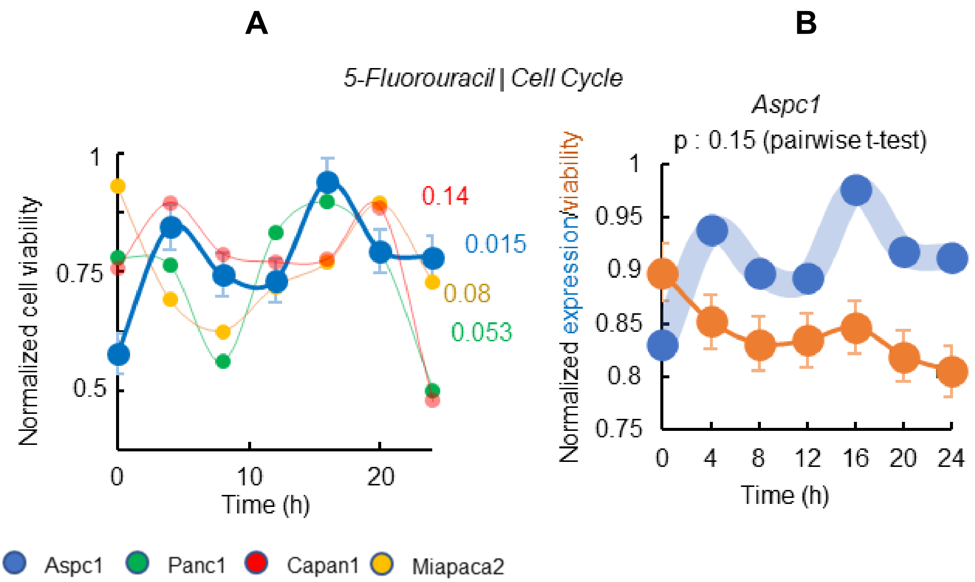

Supplementary Figure 9: Temporal drug efficacies of PDA drugs. A) B) Temporal changes in the normalized cell viability of 5-Fluorouracil (5-FU) in PDA cell lines. C) Comparison of the amplitude of composite gene expression of cell cycle hub genes and 5-FU efficacy in ASPC1 cell line. Colors represent the cell lines. Y-axis is the normalized cell viability for each line. Each datapoint represents an average of 3 replicates. p-value corresponds to the significance of the effect of time of therapy on viability of each cell line. Error bars represent standard deviation from 3 independent experiments.

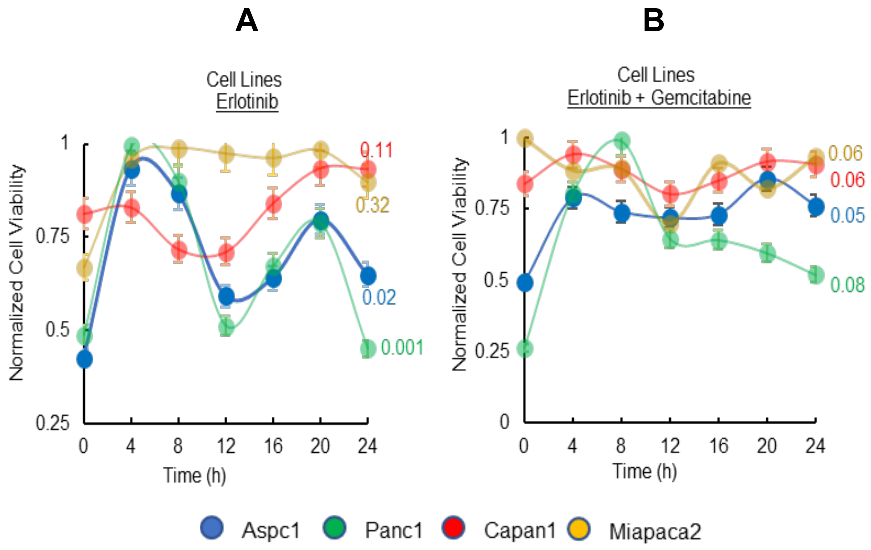

Supplementary Figure 10: Temporal drug efficacies for A) Erlotinib and B) Erlotinib + Gemcitabine in 4 cell lines. Colors represent the cell lines. Y-axis is the normalized cell viability for each line. Each datapoint represents an average of 3 replicates. p-value corresponds to the significance of the effect of time of therapy on viability of each cell line. Error bars represent standard deviation from 3 independent experiments.
